# Integrating the Ecosystem Services Framework to Define Dysbiosis of the Breastfed Infant Gut: The Role of *B. infantis* and Human Milk Oligosaccharides

**DOI:** 10.3389/fnut.2020.00033

**Published:** 2020-04-14

**Authors:** Rebbeca M. Duar, Bethany M. Henrick, Giorgio Casaburi, Steven A. Frese

**Affiliations:** ^1^Evolve BioSystems, Inc., Davis, CA, United States; ^2^Department of Food Science and Technology, University of Nebraska, Lincoln, NE, United States

**Keywords:** gut microbiome, dysbiosis, human milk oligosaccharides, ecosystem services, microbiome modification, microbial ecology, symbiosis, probiotics

## Abstract

Mounting evidence supports a connection between the composition of the infant gut microbiome and long-term health. In fact, aberrant microbiome compositions during key developmental windows in early life are associated with increased disease risk; therefore, making pertinent modifications to the microbiome during infancy offers significant promise to improve human health. There is growing support for integrating the concept of ecosystem services (the provision of benefits from ecosystems to humans) in linking specific microbiome functions to human well-being. This framework is widely applied in conservation efforts of macro-ecosystems and offers a systematic approach to guide restoration actions aimed to recover critical ecological functions. The aim of this work is to apply the ecosystem services framework to integrate recent studies demonstrating stable alteration of the gut microbiome of breastfed infants when *Bifidobacterium longum* subsp. *infantis* EVC001, a gut symbiont capable of efficiently utilizing human milk oligosaccharides into organic acids that are beneficial for the infant and lower intestinal pH, is reintroduced. Additionally, using examples from the literature we illustrate how the absence of *B. infantis* results in diminished ecosystem services, which may be associated with health consequences related to immune and metabolic disorders. Finally, we propose a model by which infant gut dysbiosis can be defined as a reduction in ecosystem services supplied to the host by the gut microbiome rather than merely changes in diversity or taxonomic composition. Given the increased interest in targeted microbiome modification therapies to decrease acute and chronic disease risk, the model presented here provides a framework to assess the effectiveness of such strategies from a host-centered perspective.

## Introduction

Disruption to the composition and function of the early life gut microbiome is now recognized for its role in irregular immune development (1, 2), metabolic disorders (3) and inflammation (4, 5). Several of these phenotypes have been reconstructed using animal models or epidemiological approaches providing a compelling link between aberrant microbiome development in early life and these negative health outcomes (6–9). Thus, if pandemic non-communicable diseases such as type 1 diabetes, obesity, allergy, and asthma are associated with impaired microbiomes during infancy, as suggested by emerging evidence (3, 10–15), then relevant modulation of the microbiome in early life provides a compelling solution for addressing the increasing public health burden associated with these diseases. However, evaluative parameters to identify desirable microbiome compositions and their potential interrelationship with health, are currently lacking.

The application of methods derived from ecological theory and evolutionary biology have been fundamental to elucidating the factors that shape the microbiome throughout the lifespan. In this work, we apply concepts from the “ecosystem services” framework (16) to guide the ecological assessment of the breastfed infant gut microbiome from a host-centered perspective. We first describe the ecological processes that shape and define the composition of the microbiome in early life. This description is centered on the hypothesis that human hosts select, via diet (human milk), for the enrichment of specialized symbionts that fulfill beneficial functions underlying the provision of ecosystem services that contribute to their fitness and well-being. We then propose a model in which the absence of these beneficial functions and the consequential reduction in one or more ecosystem services can be defined as dysbiosis. To demonstrate the applicability of the model, the discussion is centered on the coevolution of specialized bifidobacteria, namely *B. infantis*, for which clinical evidence is available (17). Finally, we summarize, as evidence for this model, large cohort studies indicating the absence of bifidobacteria in early life is associated with negative health outcomes.

## Ecological Processes Shaping the Composition of the Breastfed Infant Gut Microbiome

**Figure 1:**
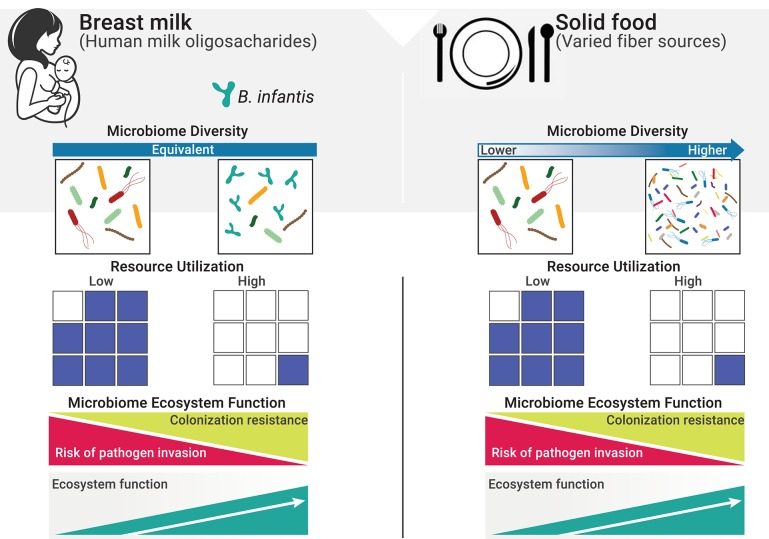
Resource utilization and diversity in the gut in determining invasion resistance. The resource landscape in the human gut is vastly influenced by diet. One fundamental function of the microbiome is to keep potential pathogens at bay by direct competition for space and resources (i.e., co-lonization resistance, a regulating service). Excess resources (blue squares) represent open niche opportunities and increase the risk of colonization by invasive species, including pathogens. When resources are efficiently utilized, the risk of successful invasion is greatly reduced due to the lack of available resources to sustain growth (28). On a solid food diet, a more diverse composite of species is required to deplete the greater variety of resources reaching the gut (29). However, in early life, and while diet is restricted to mother's milk, resource utilization is independent from diversity and will only reach maximum levels when specialized species able to efficiently consume HMOs are present.

Immediately following birth, the neonatal intestine becomes rapidly colonized by microbes from the mother and the surrounding environment. Infants delivered by cesarean section are more likely to become colonized by environmental microorganisms from the maternal skin, healthcare staff and hospital surfaces. Vaginally delivered infants come in contact with bacteria from mother's vaginal canal and the fecal microbiota (18, 19). From this initial load of microbes, the allochthonous, vaginally-derived and environmental species are then rapidly replaced by organisms adapted to the gut (20–23); however, the microbiome differences based on delivery mode persist over time (19). Nutritional resources that reach the gut are another major factor influencing the neonatal gut microbiome, in terms of both composition and function. In exclusively breastfed infants, human milk oligosaccharides (HMOs) represent the main nutritional resources for bacteria in the gut. As a result, the gut microbiome of exclusively breastfed infants exhibits lower alpha diversity and higher abundance of specialized taxa able to metabolize HMOs, namely bifidobacteria (24–27). In the absence of specialized infant-associated species of bifidobacteria, HMOs are under-utilized, resulting in excess resources with profound impacts on ecosystem function (Figure 1). Cessation of breastfeeding and the introduction of solid foods represent a major shift in the nutritional resource landscape and a more functionally complex community of microbes is then required to deplete the greater variety of dietary substrates reaching the large intestine (Figure 1).

Additional ecological events, including random processes, ultimately influence the overall composition of the infant gut microbiome; however, initial microbiome inoculation based on birth mode, and the subsequent environmental selection through the provision of selective substrates from human milk, are the two major ecological processes shaping the gut microbiome of breastfed infants (30–33).

## Biological Considerations in Defining a Healthy Infant Gut Microbiome

Identifying a healthy gut microbiome in both infants and adults has proven to be a major challenge to the scientific and medical fields (34). Historically, diversity has been speculated to maximize functionality, in a generalization of the “insurance hypothesis” (35–38). However, diversity indices are of limited value alone and have proven insufficient to determine ecosystem functionality, or to categorize microbial ecosystems as healthy or unhealthy (37, 39) (see Box 1 for an in-depth discussion on the limitations of diversity). Moreover, taxonomic composition can be highly variable among individuals, while functions encoded by the gut microbiome are remarkably coherent (45) and breastfed infants across different geographies develop a common microbial functional core (15, 32, 33). This implies hosts are under a strong pressure to select high-fidelity microbial partners to maintain key ecosystem functions (38), and that breast milk establishes key niches that can only be occupied by specialized taxa (46) (Figure 1). Furthermore, given the host and its microbiome operate as a highly interconnected and co-evolved ecosystem in which interactions among members and community characteristics are governed by the principles of community ecology, we argue the evaluation of gut microbiomes can only be successful if based on ecological and evolutionary criteria. To this end, the ecosystem services framework has been implemented to link ecological processes of macro-ecosystems with elements of human well-being (47) and has recently been adapted to value the services of gut microbial ecosystems from a host-centered perspective (16, 48, Box 2). Therefore, we propose to use this framework to guide the assessment of the infant gut microbiome and to determine the ecological conditions within the gut that may increase host health and, ultimately, fitness.

Box 1 **Diversity: How is it measured and what does it mean?**

There are two main types of diversity computed in microbial ecology studies, particularly as it pertains to microbiome profiling: alpha diversity and beta diversity.

**Figure 2:**
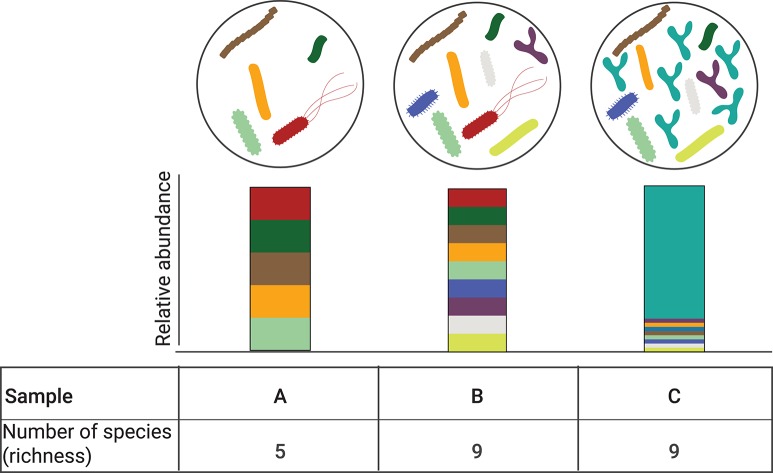
Alpha diversity is independent of relative abundance. Three different bacterial communities are depicted (A–C). Corresponding relative abundance of the individual species in the bacterial comminutes is represented by the stacked bar graphs. Bacterial communities B and C have the same number of observed species (*n* = 9) but their relative abundance is different, with community C being dominated by one species. While the alpha diversity can be computed with different metrics, when accounting for community richness, communities B and C species have the same alpha diversity.

**Alpha diversity** refers to the measure of diversity within a specific ecological community or locality in a given sample. Depending on the metric used, this index describes either species richness (i.e., the number of different species in a community); or both species richness and the evenness (i.e., the distribution of the species' abundances in the community) (40). There are several metrics to determine alpha diversity, each different in their sensitivity to richness and evenness (41). Depending on the index used, it is possible that no change in alpha diversity may be detected despite the presence of highly divergent community compositions (Figure 2). **Beta diversity** is a measure of diversity between samples. It answers the questions: How different is the microbial composition in one sample or group of samples compared to others? How many species are shared between samples? Similar to alpha diversity, there are different metrics to establish beta diversity. Some methods are purely qualitative based on presence/absence of species, while others include a quantitative component and take into account a phylogenetic distance between species. Each method presents its own inherent biases and sensitivity capturing changes in community composition.

**Uses and limitations of diversity in microbial ecology**

Diversity is speculated to maximize the functionality in a generalization of the “insurance hypothesis” (35, 36), which suggests that stabilization of communities against decline in function is improved by increasing diversity (42, 43). Thus, higher diversity is often assumed to be desirable. However, unless substantial functional redundancy exists in a microbial community, any loss in key functional species will likely alter the capacity of the microbiome to support ecosystem services (44). Further, a reduction in diversity is not necessarily unfavorable to the host, especially when it is a consequence of the selective enrichment of health-promoting symbionts.

Another inherent challenge exists in the lack of an accepted, absolute value of diversity for a given community. Moreover, as previously discussed [see (39) for an excellent discussion on the matter], diversity is relative and always constrained by method of measurement. In fact, different indices vary in their sensitivity to species richness and evenness, and inferences made can differ widely depending on the measure chosen. Thus, caution must be exercised when drawing conclusions from any one diversity index and when comparing findings across studies.

Overall, simplifying the microbiome to a measure of biodiversity has obvious limitations as it does not reflect composition or function, or relevant ecosystem properties such as stability, productivity or invisibility. We and others (37, 39) argue that the continued use of this index, without context of function, distracts the field from the development of relevant hypotheses to gain insight into the underlying ecological mechanisms driving patterns and processes in microbial communities and their potential relationship to host health.

Box 2 **Advantages of an ecosystem services paradigm to evaluate the infant gut microbiome.**

The application of concepts drawn from applied macroecology research has provided important insights into the mechanisms shaping the gut microbiome, especially as it relates to how microbial communities assemble, function and evolve (38, 49–53), and how these processes influence human health (54, 55).

Unlike abiotic geographies for macroecology, hosts have faced millennia of coevolution to shape the populations of microbes that colonize them. Exquisitely specific mechanisms to select for specific microbial symbionts have been described for plants (56) invertebrates (squid, insects) (57), and vertebrates (58–60). Selective pressures have shaped these interactions between host and microbe over time, and in the gut microbiome, toward selection for the key ecosystem services that improve host health (i.e., fitness). Evaluation of the infant gut microbiome through the lens of ecosystem services will facilitate the identification of key ecosystem “service providers” as those species whose functions are critical for the delivery of a given service. Colonization resistance and access to specialized foods or diets (provisioning services) are examples of ecosystem services where research may offer clues as to how services in the gut microbiome have been maximized by host-microbe interactions under strong selective pressures.

The ecosystem services framework is widely applied to evaluate terrestrial and marine ecosystems (47) and was recently adapted to evaluate the mammalian gut (16). Viewed through the lens of ecosystems services, the goods and services humans obtain from their microbiomes can be categorized as supporting, provisioning, or regulating (Table 1). Provisioning services are those obtained directly from the production of goods, e.g., microbial production of vitamins, antimicrobials, organic and short-chain fatty acids. Regulating services are those involved in maintaining stable ecosystem conditions, e.g., resistance to pathogen invasion. Supporting services are those necessary for the production and maintenance of all other ecosystem services, e.g., generation of pioneer products.

One main advantage of applying the ecosystem service model to evaluate the infant gut microbiome is that it facilitates the systematic identification of key “service providers” whose functional traits underpin the delivery of a given service (61, 62). By explicitly linking functional traits to ecosystem service delivery, it is possible to assign “functional importance” and “irreplaceability” indices, and correspondingly, predict the extent to which the loss of key “service provider” species can impact the ecological processes that sustain ecosystem functioning (35, 62).

**Table 1:** Ecosystem services, functions, traits, measures, and dysfunction consequences.

Ecosystem service^*^	**Description of benefit**	**Underpinning functional traits of ecosystem service providers**	**Measures of ecosystem's functionality**	**Consequences of ecosystem dysfunction**
**Supporting** Basic ecosystem processes that maintain the generation of all other services	Generation of pioneer products (primary production)	Capacity to stably colonize and generate pioneer products efficiently from the available ecosystem resources	Bioconversion rate	Decreased ecosystem functions
**Provisioning** Products, nutritional compounds and energetic outputs from ecosystems	Recovery of energy from non-digestible/absorbable substrates from the host's diet	Ability to efficiently access and metabolize the available resources (i.e., HMOs)	Production of organic acids and bacterial biomass from fermentation of HMOs Residual HMOs in stool.	Inefficient resource utilization. Loss of HMOs in the stool
**Regulating** Moderation and maintenance of essential ecological and conditions and processes.	Resistance to invasive species and prevention of pathogenic overgrowth Maintenance of mucosal and epithelial integrity	Establish abundant and stable populations. Effectively deplete the utilize the available resources (i.e., HMOs) without cross-feeding Reduce intestinal pH through the production of organic acids	Ecosystem stability index Mucosal and epithelial barrier integrity Fecal pH	Increased vulnerability to invasion and/or to the overgrowth of virulent and antibiotic resistant gene-carrying bacteria Elevated endotoxin levels Overgrowth of mucolytic bacteria

* *As outlined in by McKenney et al. (16) according to criteria established in the Millennium Ecosystem Assessment (47)*.

In the following sections we outline three key ecosystem services: (1) supporting services; (2) provisioning services; and (3) regulating services underlying the relationship between the breastfed infant and the gut microbiome based on previously defined criteria (16). Specifically, we discuss the ability of *B. infantis* to efficiently utilize resources (i.e., HMOs) and produce organic acids as key functional traits that sustain the provision of these services.

## Supporting and Provisioning Services of the Gut Microbiome in Early Life

Organic acids, including short chain fatty acids (SCFA), are the major metabolic products of anaerobic microbial fermentation in the gut and have demonstrated roles in human health (63, 64). In the breastfed infant gut, fermentation of HMOs into lactate and acetate depends critically on specialized primary degrader organisms that have the metabolic machinery to capture and metabolize these complex compounds (46). This process generates pioneer products (supporting service; Table 1) and releases energy that is otherwise inaccessible to the infant (provisioning service; Table 1). Selected strains of bifidobacteria and *Bacteroides* metabolize HMOs (24), but only *B. infantis* contains complete pathways enabling intracellular HMO-transport and degradation. Consequently, it is the only organism with the demonstrated capacity to significantly increase the production of lactate and acetate in the breastfed infant gut while simultaneously decreasing residual HMOs in the stool of breastfed infants (17, 65). In fact, in the absence of *B. infantis*, high concentrations of these HMOs are expelled into the stools of infants (1, 17, 66–68) which is a clear indication of low utilization of these resources (i.e., HMOs) in the gut, even when compared to infants colonized by other bifidobacteria. This observation highlights the importance of *B. infantis* in providing and provisioning services that underlie the overall function of the infant gut microbiome ecosystem (Table 1).

In addition to their role as pioneer substrates in the gut, organic acids and SCFA can enter circulation and directly affect the adipose tissue, brain, and liver (69–71). Acetate has been proposed to have an important role in inducing anti-inflammatory effects via the modulation of regulatory T-cells and anti-inflammatory cytokines (70), as well as improve mucosal epithelial integrity in the gut leading to protection from infectious disease in animal models (64). Lactate crosses the blood-brain barrier and functions as a modulator of neural activity, and is actively transported by gut epithelial cells (72–74). Acetate and lactate are also precursors of butyrate, which has anti-tumorigenic and anti-inflammatory properties and provides energy to gut epithelial cells (64). Overall, these microbially-produced organic acids have a major influence on host physiology. Thus, the presence of taxa able to efficiently metabolize HMOs into these key metabolites is critical to the delivery of fundamental ecosystem services that can affect the short- and long-term health of the growing infant.

## Colonization Resistance and Stability are Critical Regulating Services of the Infant Gut Microbiome

**Figure 3:**
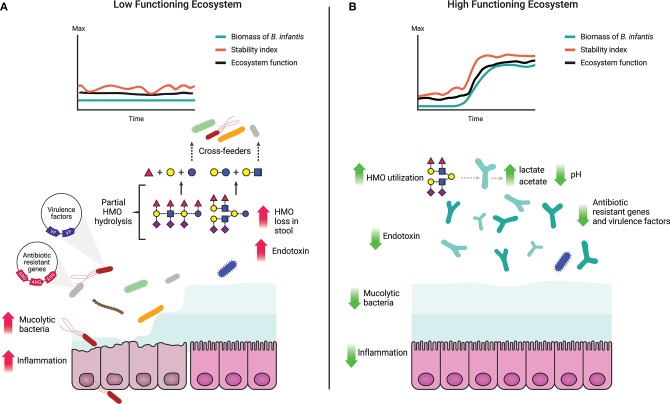
Characteristics of a high and a low functioning infant gut microbiome based on delivery of ecosystem services. **(A)** A low functioning infant gut microbiome ecosystem where the resources (HMOs) are inefficiently utilized leading to their loss in the stool (17) and potential cross-feeding to non-adapted opportunistic taxa. Prevalence of non-adapted opportunistic taxa leads to loss of ecosystem stability and decreased ecosystem function along with increased abundance of virulence factors and antibiotic resistance genes (82, 84, 85). Overgrowth of mucus-degrading bacteria and elevated levels of endotoxin compromise the intestinal barrier leading to chronic enteric inflammation and/or increased susceptibility to bacterial translocation (5, 89, 90). **(B)** A high functioning gut microbiome where ecosystem functions and stability are maintained over time, corollary to an increase in biomass of *B. infantis*. HMOs are efficiently utilized by *B. infantis* and converted into cell biomass, organic and SCFA. Production of organic and SCFA reduces the luminal pH (17) creating an unfavorable environment for opportunistic taxa, including virulent, antibiotic resistant, and mucolytic bacteria.

One of the critical functions of the gut microbiome is to protect the immunologically naïve infant from acquiring exogenous pathogens and to prevent the overgrowth of opportunistic commensals (10, 75), a process known as colonization resistance (76, 77). Direct competition for resources, metabolic exclusion by production of organic acids, and indirect stimulation of the mucosal barrier system are well-characterized mechanisms by which the microbiome provides the host with this regulatory service (78). More competition for resources increases ecological stability at the expense of diversity by favoring the growth of specialized taxa, and limits the ability of invading microbes to establish and replicate (79). Thus, increased stability is central to the delivery of this regulatory service (Table 1) as stable ecosystems are inherently more resistant to external disturbances (42). In a clinical study, it was shown that colonization with *B. infantis* EVC001 significantly increases the stability of the infant microbiome (17). Moreover, consumption of HMOs by *B. infantis* produces acidic end-products mainly lactate and acetate, thereby altering the intestinal environment to prohibit the growth of pH-sensitive populations (e.g., *Enterobacteriaceae* and *Clostridia*) (69, 80, 81) including known enteric pathogens (17, 82), many of which carry antibiotic resistant genes (83–85). Further, the resulting high abundance of bifidobacteria contributes to maintaining intestinal barrier function through the production of acetate and tryptophan metabolites, and the reduction of mucus-eroding bacteria (86–89). Thus, the regulating services infants obtain from a microbiome abundantly colonized by *B. infantis* represents an archetypal model of protection, in which the host selects (via HMOs) microbial taxa most adept at strengthening epithelial defenses as well as creating biotic (i.e., competition for resources) and abiotic (i.e., pH) resistance barriers against invasion (28). A conceptual depiction of these concepts is shown in Figures 1, 3.

## Integrating Ecosystem Services in the Evaluation of the Infant Gut Microbiome

The application of traditional concepts from macroecology has proven successful in providing relevant insight into the ecological dynamics that govern the human microbiome (38, 49). According to ecological theory, ecosystem productivity can be measured by total biomass and by changes in the concentration of a limiting substrate (35, 62, 91). In the gut, dietary and host-derived carbohydrates are the primary resources for microbial metabolism (31, 32). Productivity of the ecosystem can thus be determined based on the efficiency of their utilization, and in concert, determining bacterial biomass (Table 1; Figure 3). Together, these two functions offer complementary and independent approaches to monitor productivity and to identify states in which the delivery of the ecosystem services is maximized. Thus, by combining evaluations of ecosystem productivity and the generation of ecosystem services we propose a model for the definition of dysbiosis of the breastfed infant gut as a low-functioning ecosystem, in which the gut microbiome community is characterized by (1) low stability even without perturbations (e.g., diet change or antibiotics); (2) high susceptibility to invasion by external taxa; and (3) low utilization of the available resources (i.e., HMOs). The alternative to dysbiosis or a “healthy” state is characterized as being a high-functioning ecosystem when the gut microbiome community is: (1) stable over time, (2) resistant to invasion by allochthonous bacteria; and (3) shown to exhibit a high conversion of HMOs to pioneer products and biomass of benefit to the host (Figure 3). Overall, by focusing on function, this model is agnostic to method and index of choice and provides a quantifiable and objective approach to evaluate the microbiome.

## Application of the Model to Evaluate the Symbiosis Between *B. Infantis* and the Breastfed Infant

Humans live in symbiosis with the composite of microbial inhabitants residing in their intestinal tracts but the contribution of specific species to the overall ecosystem function and terms of the individual symbiotic relationships, which can range from commensal to parasitic, are less understood. Considering the ecosystem services the infant host obtains from selectively favoring the growth of *B. infantis*, it is evident that the symbiotic relationship is mutualistic. Free selectively consumed resources like HMOs are extremely rare in nature and the composition of HMOs is unique among mammals (92, 93). Of the thousands of species able to colonize the human gut, only a very limited number of species have the molecular machinery to utilize them (24). Within the genus bifidobacteria, only *B. infantis* encodes the complete set of genes required to transport, and intracellularly deconstruct and metabolize all the chemical structures found among HMOs (65), thus indicating maintenance of these genes is under strong selection. Indeed, since its discovery, *B*. *infantis* has so far been exclusively found in association with human beings (94) and phylogenetic analysis indicates humans and bifidobacteria have co-speciated (95). Taken together, the association of *B. infantis* and the breastfed infant host presents strong characteristics of an exclusive symbiotic alliance that has persisted over evolutionary timescales, whereby the human host requires the symbiont to access a significant portion of its diet (i.e., HMOs), while concurrently the symbiont benefits from the nutritional niche provided by the host. This concept is congruent with well-established models of coevolved symbioses (57, 96, 97).

Interdependent biological alliances are best understood in binary symbiotic models (57). One invariable lesson from decades of research in these model systems has been that aposymbiosis (i.e., the absence of the symbiont) can represent a major stressor to the host and often results in physiological and developmental deficiencies. For example in the well-characterized Squid-*Vibrio* model, external perturbations are markedly different between apo- and symbiotic squids (98, 99). Indeed, the presence of *V. fischeri* may help modulate the host stress responses (100). Similarly, the removal of nutritional symbionts (i.e., symbiotic bacteria that help their animal partners digest, absorb, and metabolize complex nutrients) is known to pose appreciable fitness costs to the host (96, 101, 102). Notably, the removal of vertically transmitted (from parent to offspring) nutritional symbionts has been shown to have the greatest negative impact on host fitness (102), which bears surprising parallels to the conspicuous depletion of *B. infantis* among infants with severe acute malnutrition (103) and with the inverse correlation between fecal pH and stunting (104). Further examples include aposymbiotic pea aphids which have reduced growth rates, attain a lower adult size, and are reproductively sterile (101) and fruit flies, for which the presence of the facultative symbiont *Lactobacillus plantarum* is critical to the growth and maturation of larvae ingesting nutritionally suboptimal diets (9). Together, these examples demonstrate broadly that the disruption of ancient symbiotic associations can have negative implications on the host.

All data indicate human infants have evolved to partner with key symbiotic gut bacteria specialized in metabolizing host-provided resources in the form of HMOs; however, it appears over time the role of *B. infantis* and the impact of its absence from the infant gut have become obscured, likely because the generational loss of *B. infantis* predates the advent of high resolution tools to investigate the gut microbiome. For instance, substantial fecal excretion of HMOs and high fecal pH are not considered abnormal, and considerable instability of the gut microbial ecosystem is considered normal in early life (33). However, historical records suggest bifidobacteria was once more prevalent among infant populations in developed nations than what contemporary reports indicate (105), and correlative evidence from large cohort studies suggest absence of this key symbiont comes with important negative acute and chronic health consequences during a critical developmental stage (2, 4, 103, 104, 106).

## What are the Acute and Chronic Health Consequences of the Absence of *B. Infantis* in the Infant Gut?

The importance of individual species to ecosystem function, and ultimately to the services, can become apparent through their loss. There is growing appreciation that interventions known to disrupt microbiome development may lead to the extinction of certain taxa across entire populations (107). Widespread antibiotic use, cesarean section delivery, and formula feeding are associated with altered gut microbiome compositions and subsequent negative health outcomes, including obesity and autoimmune diseases (3, 108, 109). In particular, the increased prevalence of these dietary and medical interventions has been associated with the decline of *Bifidobacterium* over the past century (20, 21, 60, 105, 110, 111). We pose the loss of critical functions in the gut resulting from the decline in the prevalence of *B. infantis* may have selected for microbiota that lack the resilience and stability during critical stages of immune and metabolic development. In fact, lower abundance of bifidobacteria has been associated with greater risk for developing colic, atopic dermatitis, asthma, food allergies, type I diabetes and chronic inflammation (2, 10, 11, 15, 112). Additionally, infants lacking *B. infantis* show signs of chronic enteric inflammation during the first 60 days of life (5), which has been directly linked to an increased risk of certain chronic disorders such as atopy and asthma later in life (113).

Interestingly, in geographic locations where breastfeeding rates are high and vaginal birth is widespread, *Bifidobacterium* is normally abundant in infant microbiomes (66, 114, 115). In contrast, the gut communities of infants in developed countries are largely unstable and highly variable (25, 111) and the distribution of *Bifidobacterium* is notably bimodal (26). This variation is clearly evident in a recent comparison of the gut microbiome of infants in geographically similar but developmentally diverse locations in which the level of *Bifidobacterium* was found to be higher in infants in more resource-limited locations, which correlated with decreased incidence of autoimmune and allergic diseases (2). Together these findings raise the question of whether the modern infant gut microbiome has been fundamentally altered from that of our ancestors and how the loss of key symbiotic species and the resulting disruption in immune development could be connected to the increased incidence of metabolic, autoimmune, and allergic diseases observed in developed countries today.

Fecal pH is another factor that has changed significantly over the past century and is consistent with the loss of *Bifidobacterium* (105). Fecal pH values directly correlates with the bacterial species colonizing the infant gut, particularly pertaining is the direct association between lower fecal pH and significantly decreased abundance of potentially harmful bacterial populations (i.e., *Clostridiaceae, Enterobacteriaceae, Peptosteptoccocaceae*, and *Veillonellaceae*) (105). These findings are intriguing, as an abundance of specific *Enterobacteriaceae* species induce gut inflammation (21), which has been positively associated with colic and crying in infants (116, 117). These adverse conditions may be due to the fact that *Enterobacteriaceae*-derived lipopolysaccharides induce stronger inflammatory activity compared with other lipopolysaccharide-producing bacteria (2, 118). In addition, lower fecal pH has been shown to be associated with better anthropometric growth scores (104) and improved thymic growth, a sign of immune system development (1). This may partially explain why *B. infantis*-colonized infants exhibit more robust vaccine responses (66, 119) and why there is a reduced incidence of autoimmune diseases in populations colonized with high levels of *Bifidobacterium* (2). Taken together, these data indicate functions provided by key symbiotic partners (i.e., *B. infantis*) during infancy have a strong impact on development and conversely, the absence of these taxa can have negative health consequences, underscoring the need to restore specific beneficial taxa to the infant gut.

## Relevance, Applications, and Limitations of the Model

With the growing recognition of the role of the microbiome in human health, the incorporation of microbiome-based diagnostics will inevitably become routine. In fact, a number of commercial tests are currently available to the general public and physicians are increasingly requested to interpret test reports. However, we currently lack a “gold standard” for what constitutes a healthy microbiome. Here, we proposed an anthropocentric model whereby gut microbiome function is determined in terms of ecosystem services that ultimately benefit the infant. Thus, microbiome composition can be evaluated objectively with regard to its contribution to host health, facilitating interpretation by health professionals. Furthermore, linking functional traits to specific ecosystem services may assist both the development of prognostic tools of infant microbiome function and probiotic interventions aimed at restoring the ecosystem services of the infant gut microbiome.

However, it is important to recognize that this model is limited to conditions in which the nutrient landscape in the gut is shaped by a single nutritional resource (i.e., HMOs) and will have to be re-validated for conditions known to shift the type and amount of resources as well as the distribution of biomass. Such conditions include, the introduction of complementary foods, formula feeding, antibiotic use and other microbiome-modifying practices. Moreover, the principles on which this model is based may be affected by stochastic events including niche pre-emption (i.e., “first come, first served”) driven by priority effects (120). We also recognize the overall dynamics of the infant microbiome involve complex intra and inter-species interactions which are not considered in our model. For example, the ecological relevance of *Bifidobacterium* species other than *B. infantis*, which are known to have limited capacity to metabolize HMOs but are found in the stools of infants, is currently unknown (121). Additionally, future models should aim to integrate the non-bacterial microbial inhabitants of the microbiome (e.g., virus, archaea, fungi, and other eukaryotes) which are increasingly recognized as important functional components. Nevertheless, the ecological principles presented here, can be broadly applicable to other host species, and evaluation of additional body sites, and can be adapted to inform the selection of taxa that may be relevant for health in other stages of life.

Lastly, we hope this work encourages the field to propose analogous models that incorporate ecological theory and testable frameworks to identify microbiome characteristics that are conducive to health or disease.

## Author Contributions

RD, BH, GC, and SF drafted and wrote this manuscript. All authors are responsible for idea conception, critical evaluation, and manuscript review.

### Conflict of Interest

RD, BH, GC, and SF are employed by Evolve BioSystems, Inc.

## Glossary

**Allochthonous:** An organism whose origin is different than that in which is found.

**Biomass:** Total quantity of living organisms of a species per unit area of a given habitat.

**Dysbiosis:** Vague/imprecise term often used to describe a microbiome composition that is different from the control and/or observed in subjects with a particular disease or condition. No scientific consensus has been reached on its definition. The origins and use of the term are reviewed by Hooks and O'Malley (122), and the casual obfuscation of its use to progress in the field, is discussed in Olesen and Alm (123) and Brussow (124).

**Ecosystem services:** Refers to the benefits to be gained from properly functioning ecosystems.

**Ecosystem functioning:** Refers to all processing and transport of energy and matter in an ecosystem, integrating multiple individual functions of the ecosystem including the production of biomass, the biochemical cycling of resources and the ability to resist invasion by allochthonous species.

**Fitness (ecology):** An organism's adaptation to the environment that increases its ability to propagate its genes. Genotypes with higher fitness are therefore selected for in the next generation (see natural selection). Fitness is environment-specific and directly related to the number of offspring produced.

**Insurance hypothesis:** Suggests that stabilization of communities against decline in function resulting from invasion, species loss, or fluctuations in abiotic features of the environment is improved by increasing diversity, and that diminishing fluctuations over time increases the overall productivity or services provided by the community.

**Limiting substrate:** Specific resource by which the productivity rate of an ecosystem depends on.

**Mutualistic:** Exchange of goods and services between species.

**Niche** (Hutchinsonian): Environmental conditions (biotic and abiotic) of a given habitat under which a species can persist and maintain stable populations without immigration from external sources.

**Opportunistic commensal:** Otherwise non-harming members of the gut microbiome that bloom upon a disturbance to the ecosystem and exert pathogenicity to the host.

**Primary production:** First level of nutrient generation in the tropic chain (food web).

**Productivity:** Rate at which energy is converted to biomass.

**Stability (ecology):** Measure of the temporal variability of an ecosystem, depends on its resistance to environmental change, and its rate of return to equilibrium following a perturbation (resilience).

**Stochastic:** With inherent randomness, the opposite of deterministic.

**Symbiosis** (From Greek: *sym* “with” and *biosis* “living”) long-term associations between organisms of distinct genetic makeup.
